# Studying Brains. What could neurometaphysics be to NeurotechEU?

**DOI:** 10.3389/fnins.2023.1155547

**Published:** 2023-05-25

**Authors:** Jan Bransen, Freek Oude Maatman

**Affiliations:** ^1^Philosophy Programme, Behavioural Science Institute, Faculty of Social Sciences, Radboud University, Nijmegen, Netherlands; ^2^Radboud Teaching and Learning Centre, Radboud University, Nijmegen, Netherlands; ^3^Department of Philosophy, Groningen University, Groningen, Netherlands

**Keywords:** metaphysics, neuropragmatism, neurometaphysics, continuous learning, Dewey, relational ontology, Cartesianism

## Abstract

NeurotechEU has introduced a new conceptual hierarchy for neuroscientific research and its applications along 8 different core research areas, including the so-called ‘neurometaphysics’. This paper explores this concept of neurometaphysics, its topics and its potential approach. It warns against an endemic Cartesianism in (neuro)science that somehow seems to survive explicit refutations by implicitly persisting in our conceptual scheme. Two consequences of this persisting Cartesian legacy are discussed; the isolated brain assumption and the idea that activity requires identifiable neural ‘decisions’. Neuropragmatism is introduced as offering the promise of progress in neurometaphysics, by emphasizing that (1) studying brains interact organically with their environment and (2) studying brains requires an attitude of continuous learning.

## 1. Introduction

Brains, especially human brains, are the most intricate and miraculous existences in the entire universe. The complexity of their structure, the diversity of their parts, the variety of their modes and levels of integration, and the sheer magnitude of their dynamics… it is way beyond our comprehension. And it is precisely because of this that eight European universities have decided to collaborate in the study of the brain by setting up Neurotech^EU^, the European University of Brain and Technology.

There is something deeply fascinating about studying brains, especially once we take into account that brains are the kind of entities that made study – *study* – possible at all. Does this mean that in studying the brain we meet the paradigm of reflexivity, the materialization of what Kierkegaard dazzlingly characterized as the core of ourselves, of our being human: “a relation that relates itself to itself” (Kierkegaard, 1849)? If we take this characterization seriously we are at once in the heart of metaphysics, that uncomfortable philosophical discipline often considered to be as profound as obscure. The addition of that trendy prefix “neuro-” makes this suggestion even more unintelligible. What are we talking about?

**Figure 1:**
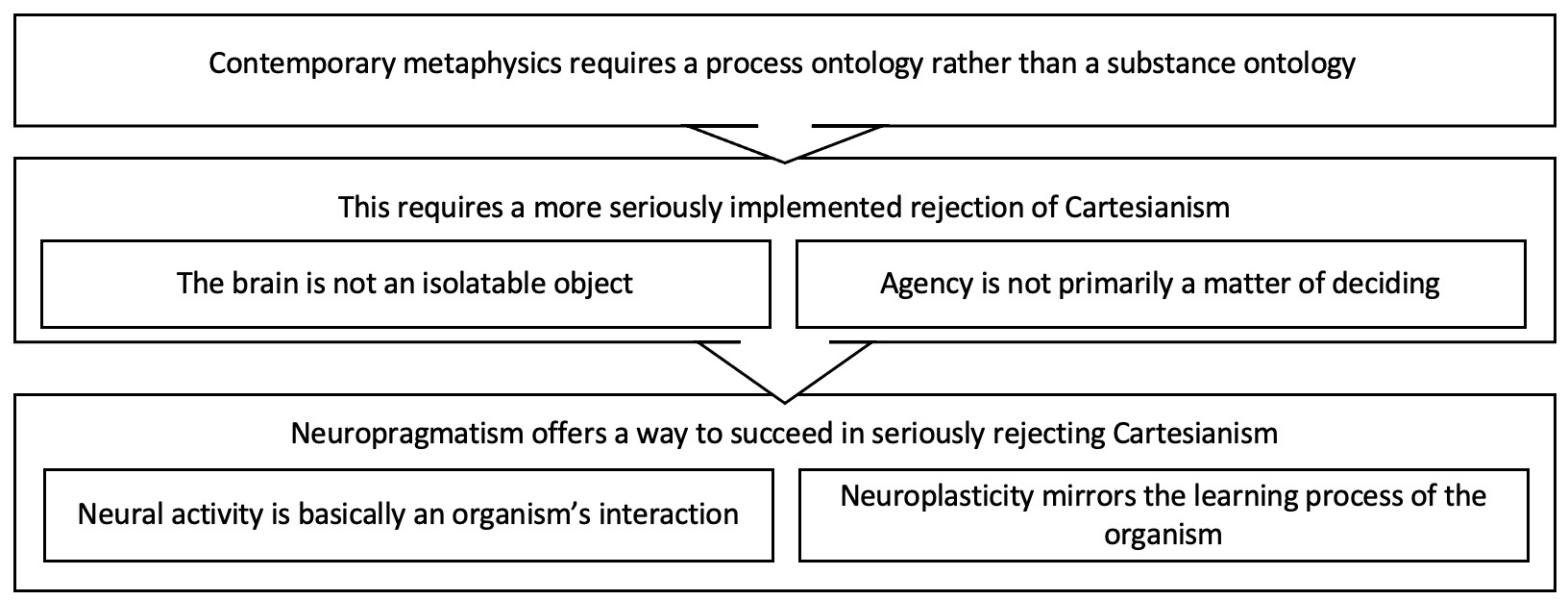
Structure of the argument.

In this paper, we argue for one possible answer to that question, which is deliberately divergent from existing approaches such as neurophilosophy and the philosophy of neuroscience (Churchland, 1986; Bickle et al., 2019). We follow up on Neurotech^EU^’s (2023) ambition to present *neurotechnology* as the most promising starting point to advance our understanding of both the structure and the function of the human brain. And we accept their identification of neurometaphysics as one of the eight dimensions of neurotechnology. This requires us to begin in section 1 with a rough indication of what we may take this term to refer to. This will invite us, in section 2, to explore a number of ways in which our contemporary understanding of science and of brains still suffers from an endemic Cartesianism. That will lead us, in section 3, to embrace the promise of neuropragmatism, spelling out the intrinsic ambiguity of the title of this paper: studying brains is a practice of people that position themselves in their environment as studying brains by positing neural patterns that mirror the inquisitive practice they constitute (Figure 1).

## 2. Thinking and being

Embarrassingly, we owe the term ‘metaphysics’ to a first century librarian, Andronicus of Rhodes, who was ordering various Aristotelian writings and who came upon a collection of treatises that did not seem to have a collective title. Since they were shelved after the books on physics he is said to have named them “*meta physica*,” “meta” meaning “after.” As Chroust (1961) has nicely argued, however, there may also be a substantial reason for this ordering, related to the Aristotelian distinction between things that are better known to us and things that are better known in themselves. From our own human intellectual or educational point of view it seems plausible to begin with studying the things that are better known to us: physical entities. But from the point of view of reality itself it seems obvious that the things that are better known in themselves are of fundamental importance. These latter things, much more difficult to study and to comprehend, constitute the subject matter of metaphysics which Aristotle himself therefore called ‘first philosophy’. We’re advised, or so Chroust argues, not to begin with ‘first philosophy’ but to first study the things better known to us and to wait with metaphysics until we sufficiently understand the physical building blocks of the universe.

There seems to be an issue with questions about the proper place of philosophy within university curricula. Philosophy courses programmed in later years tend to be assessed as scheduled too late, while courses programmed in earlier years tend to be assessed as having been scheduled too early. Hegel (1807) would understand this: it does not matter when you begin with philosophy, for it necessarily begins in the middle. Studying concepts requires that you have acquired a conceptual scheme, but this scheme then necessarily informs, and potentially deforms, your capacity to study concepts. The European University of Brain and Technology seems to follow the cautious advice of Aristotle’s editor: neurometaphysics is listed as the last dimension (NeurotechEU, 2023). But since it is included as a *dimension*, as all the other subjects are too, the setup of Neurotech^EU^ seems to resonate with the Hegelian insight: studying brains is too complex to be congruous with the one-dimensionality of a university curriculum. So let us just begin, even though it might already be too late.

Aristotle’s own interpretation of the aim of ‘first philosophy’ might be a proper starting point: “to identify the nature and structure of all that there is. Central to this project is the delineation of the categories of being. Categories are the most general or highest kinds under which anything that exists falls” (Loux, 2017). What does this mean for *neurometaphysics*? Note that Aristotle’s interpretation is compatible with the belief that metaphysics is merely possible as a human enterprise. Determining the nature and structure of all that there is, is something that *we do*, something that is as much a matter of making as it is of finding. For us, human intelligences, the distinction between things that are better known to us and things that are better known in themselves is itself fundamentally beyond intelligibility. Nevertheless, we can make good sense of what it would mean to delineate the categories of being. We do know what it would mean to understand the difference between thinking and being, between the thought of an object and the reality of that object, completely independently of what we think about it. This makes good sense, even if we would not know how to attain the truth in this area, and even if it would turn out to be beyond the limits of our understanding to make sense of there being *truth* in this area at all.

For a long time the categories of ‘substance’ and ‘property’ have been assumed to be basic (Van Inwagen and Sullivan, 2021). And despite Kant’s critique of the transcendental claims of traditional metaphysics, it seems plausible to maintain that common sense still takes these categories for granted. Whatever you think of the nature and structure of all there is, there simply are things, objects, entities, items, existences that subsist in themselves. There are dogs and chairs and stones, and these things have properties: weight and size and colour. And, yes, obviously, these things can be decomposed, and so can their parts, as well as their parts, and theirs, and so on. You can go all the way, but somehow it seems evident that we will end up with particles, somethings, substances, items that can be pointed out as the fundamental building blocks of what there is.

But something baffling happens along the way with the properties that from the commonsensical point of view seemed obviously to be equally basic as the substances. Somehow, however, properties seem to disappear when we decompose the middle-sized objects of our natural world. Dogs have weight, size and colour, but those properties seem to lose their reality on the level of their parts, especially when we are talking about their parts on the molecular, atomic or even sub-atomic level. In response, we have learned to talk about emergent properties, properties that do not exist on the lowest level but that somehow come into existence on a higher level. Apart from what this means for the reality of properties, this raises thorny metaphysical questions about the reality of levels. Do levels exist *in themselves*? Is the world a layered world, or are levels merely implications of our attempts to study and understand reality (Kim, 2002)? Such are the questions that have played a major role in the history of metaphysics. What is the reality of properties such as “redness,” “doghood,” or “being alive”? Are they features of the nature and structure of reality? Or are they mere figments of our imagination, features of the language that we use to talk about what there is?

A significant change in thinking about substances and properties took place in the beginning of the twentieth century, in an early struggle with Einstein’s discovery of special relativity, a change strikingly characterized in Ernst Cassirer’s *Substance and Function* (Cassirer, 1910). Cassirer argues that developments in science, mathematics and logic have opened up a radically new way of thinking about the nature and structure of all there is. Rather than conceiving of reality in terms of items, things or substances that take up space and that are related in all kinds of physical ways, we should conceive of all there is in terms of the relations themselves. That is, *relation* or *function* can be conceived as being the most fundamental category. More recently, this idea has been further developed and formalized under the nomer of ontic structural realism, which takes these relations and their structure to be all that is real (*cf.* Ladyman, 1998; Ladyman et al., 2007).

This provides us on the one hand with a way to conceive of all there is in the purely formal terms of mathematical physics, liberated completely from the phenomenological limits of human experience. Thinking of reality in terms of relations, allows us, for instance, to think of matter as energy, as a movement not even of particles, but of magnitudes that change place, where places are purely formally identified too, as relations between mathematical points in space–time. The purely mathematical language of functions opens up a way to conceive of the nature and structure of all that there is that is completely independent of our human historical trajectory, the developmental and evolutionary trajectory that began with thinking of things that are better known to us. It is as if we can now escape from this history: the language of mathematical functions gives us a glimpse of reality in itself, a universe in which everything is fundamentally relational.

But on the other hand it is precisely the fundamental relational nature and structure of all that there is, that shows science itself to be an intrinsically human activity. Science is a relational endeavour too, the human practice of trying to separate two relata that are intrinsically and essentially related: the act of measuring and what is measured. Science is the practice of positing the reality of what is measured with increasing distinctness by constructing it out of what we measure (Cassirer, 1910, 1944). Science itself, as a practice that implies the positing of independent existences, manifests in its own attempt to measure what can be measured, that relations are metaphysically prior to the relata. That is why neuroscience invokes neurometaphysics, the point being that the reality of brains to be studied as existing objects is a relatum that is essentially connected to another relatum, namely the reality of human beings studying brains, i.e., people engaged in neuroscience. These two are real only as relata, as extrapolations of what is essentially a relation, the relation of studying brains studying brains. Note the Kierkegaardian reflexivity.

Along these relational lines sense can be made of Neurotech^EU^’s decision to use the term ‘neurometaphysics’ for the reflective investigations that do concern the relationship between the brain as an object of study and the variety of human activities that are involved in studying the brain: neurophilosophy, neurolaw, neuroethics, neuroaesthetics, neurodesign. Studying the nature and structure of the brain in itself means studying the concepts, methods and skills that allow us to measure the brain’s activities as functional endeavours to relate itself effectively and sustainably to the complex environment in which it has to survive. Neurometaphysics captures this all in its reflective mode of studying studying.

## 3. An endemic cartesianism

Now that we have an idea of what neurometaphysics might be, we should like to proceed by first reducing the enthusiasm and optimism that seems to befit the establishment of a new European university, but that at the same time can tempt us to underestimate the enormous challenge we face in doing neurometaphysics. That is, we have to remind Neurotech^EU^ that they commit a *pars pro toto* fallacy in their mission statement by asserting that “as the product of evolution, the brain is marvellously effective in surviving in complex, partially unknown environments.^1^” For obviously the brain, being an organ, cannot survive without the body at all. Obliviously assuming it can or that it is the most crucial of all organs to this end, is most likely an echo of an endemic Cartesianism, which is apparently extremely difficult to overcome, even though almost every neuroscientist will explicitly and duly affirm that Cartesianism is dead and long since vanquished. Nevertheless, John Dewey’s observation is even after almost hundred years still easily ignored, with dreadful consequences:

To see the organism *in* nature, the nervous system in the organism, the brain in the nervous system, the cortex in the brain is the answer to the problems which haunt philosophy. And when thus seen they will be seen to be *in*, not as marbles are in a box but as events are in history, in a moving, growing never finished process (Dewey, 1925, p. 295).

We will here focus on two implicitly Cartesian presuppositions that threaten the study of brains: (1) the independent status of the mind/brain and (2) the deliberative account of agential activity.

### 3.1. The isolated brain

Cartesianism is assumed to refer most crudely to dualism, to the idea that the mind or soul is a substance entirely different from the body. The twentieth century has generated severe opposition to this mind–body dualism in the form of arguments in favor of materialism, behaviorism and functionalism, due to which the contemporary default position seems to be some kind of physicalism. According to physicalism, mental properties are either identical to physical properties or are supervenient or emergent properties, properties that can completely be accounted for in terms of relations between purely physical states. These purely physical states are generally understood to be neural states, in line with the commonly at least implicitly accepted mind/brain identity theory (Smart, 2022).

Equating the mind with the brain, however, seems to give the brain a highly special place in nature, duplicating in a variety of senses the dualism it intended to overcome. One variety of this brain–body dualism is identified by Vidal (2009) as “brainhood,” the peculiar view that we are most essentially our brain, implying, for instance, the intuition that if brain transplantation would become possible it would actually amount to a brain receiving a new body rather than the other way around. A related but different dualism is identified by Mudrik and Maoz (2014) in the use of the double-subject fallacy, as for instance in Gazzaniga’s (2006) remark that “the brain knows our decisions before we do.” Yet another brain–body dualism haunts psychiatry and psychology by the often taken for granted assumption that mental illness is a disease of the brain, i.e., consists in the disfunctioning of one of our organs (Fuchs, 2005; Lewis, 2015).

Thinking of the brain as an independent entity that can be studied, analyzed and described in isolation is merely conceivable for those who implicitly take for granted some kind of substance ontology. One must, after all, then perceive the brain as an isolated object with studiable properties, phenomena and functions that are genuinely and uniquely its own – such as the aforementioned ability to “survive in complex, partially unknown environments” (NeurotechEU, 2023). But once we would seriously try to isolate the brain and still would think of studying its function, its workings, we would quite quickly get stuck. For the brain is enmeshed in a large-scale network that extends at different levels way beyond what seem to be the organ’s physical boundaries. And as Dewey already suggested, we should consider “the cortex within the brain.” We cannot forget that the brain *itself* also is a deeply enmeshed, large-scale network, making it similarly problematic to study its parts, or parts of these parts, in isolation of the remainder of the brain – and by extension, in isolation of the body and the environment (Anderson, 2014; Pessoa, 2022). Taking this seriously, that is, taking seriously that the brain and its parts are to be identified by their functions *relative* to their broader embedding, will become much easier when we abandon a substance metaphysics. But this also means that the study of brains amounts to the study of organisms *in* nature, an intellectual endeavour that is deeply at odds with positing the existence of isolated brains.

### 3.2. The deliberative account of agential activity

Even if we would endorse the conclusion that studying brains means studying intelligent organisms and focus on how their internal makeup contributes to their successful existence *in* nature, our Cartesian legacy might still tempt us to ask the wrong questions. Wittgenstein famously asked: “What is left over if I subtract the fact that my arm went up from the fact that I raised my arm” (Wittgenstein, 1953, §622)? This question aims at distinguishing between events and actions; which movements are ‘mine’ and which merely happen? Dualists will quickly suggest an answer. A rising arm is a mere happening, a physical event like a leaf blowing in the wind, water flowing down or a pinball bouncing through a pinball machine. But when *I* raise *my* arm, this means that my mind decided to raise my arm, and it is this mental event that causes the rising of my arm. In the aftermath of Cartesianism the natural response to Wittgenstein’s question therefore might seem to be that there should be some further, causally efficacious fact that explains the difference between actions and events. What fact? What is the ‘agential activity’ that turns my arm rising into an arm raising (Kasafanas, 2011)?

Given that neuroscience has passed substance dualism by in favor of materialist views of the mind, the challenge might seem to be that we need to identify some kind of neurophysiological phenomenon that is functionally equivalent to a causally efficacious intention. This neurophysiological phenomenon furthermore must differ from other kinds of physical phenomena that cause mere bodily events that are not actions, such as reflexes. Such a functionalist understanding of the problem of action became quite popular thanks to the groundbreaking work of Davidson (1963). It is an understanding that might seem to fit with the cognitivist picture of the mind/brain as an information-processing organ that mediates between incoming perceptual stimuli and outgoing motor responses. It is a picture that emphasizes that certain kinds of neural events cause bodily movements *that are actions* – that is, movements that can rightfully be characterized as caused by supposedly conscious, intentional, rational and deliberative states of mind. This account might call the in psychology still rather popular dual process theories to mind (Strack and Deutsch, 2004; Kahneman, 2011), which suggest the existence of an impulsive and a reflexive, deliberative sytem.

Looking for those neural processes that possess the appropriate, action-generating features might, however, cause us to set off on the wrong foot (Dennett, 1987). Our point is not just that such an endeavour will entangle us in category mistakes, but mainly that it will entice us into believing that agential activity is a matter of deliberate decision-making (Levy and Bayne, 2004; Kasafanas, 2011). This suggests that acting is fundamentally a matter of deciding and that the embodiment of the mind amounts to the nexus between brain and body. Furthermore, on this viewthe automaticity caused by perception-action coupling characteristic of habitual behaviour complicates our understanding of whether or not habitual behaviour is agential.

Our point thus is not that agency cannot be attributed to neural activity. Instead, we argue that endemic Cartesianism suggests that agency should be conceived of as consisting in the presence of a volitional, decision-like neural event that is related to the actual bodily movements as cause to effect. This causal picture is what Wittgenstein’s question is challenging, an observation missed by most neuroscientific investigations of the phenomenon of free agency, whether or not they focus on conscious control (Libet 1999; Wegner, 2002; Burns and Bechara, 2007; Lavazza, 2016).

Agency, for instance the agency of neuroscientists studying brains, is not a matter of bodily movements brought about by neurally realized instances of decision-making. The focus on decision-making, deliberation or even isolated causes for action is a lagging heritage of Cartesian dualism, like the focus on isolated brains. We suggest that it would be better for neurometaphysics, and neuroscience by extension, to truly free itself from this endemic Cartesianism. It can. And neuropragmatism will lead the way.

## 4. The promise of neuropragmatism

To revitalize, expand and strengthen John Dewey’s relevance to the philosophy of cognitive science, Solymosi (2011) coined the term ‘neuropragmatism’ – another fancy neologism with the trendy prefix “neuro” (Solymosi and Shook, 2014). The important part of the term, however, is not the prefix, but the keyword. Pragmatism is an approach to metaphysics that was developed in the United States at around the same time Cassirer argued that function or relation should be the most basic category, rather than substance. Pragmatism, especially as developed by John Dewey, fits significantly well with our exploration of neurometaphysics, because of the way in which Dewey argues for a radical integration of education, science, epistemology, and ontology as basically and most fundamentally *practices*, ways for the socially intelligent organisms that we are to flourish in their lifelong adaptive engagement with their environment.

The remainder of this paper does only allow for a rough sketch of some of the promising features of the pragmatic approach to neurometaphysics. Neuropragmatism urges neuroscientists to let their attention not be drawn away by traditional dichotomies. And there are many such dichotomies that hinder rather than support the study of brains. Thinking of ourselves as socially intelligent organisms requires, according to pragmatism, that we overcome our inclination to take the following distinctions for granted:

- Subject and object. We are studying brains studying brains, and therefore we are always and by the same token both subject and object.
- Ontology and epistemology. Any claim about what there is will at the same time be a claim about our conceptual framework. And these claims, being claims, will always remain *claims*, that is speech acts, attempts to assert in practice what is the case.
- Knowledge and action. Scientists have familiarized themselves with a firm distinction between theory and practice, attached as they are to the spectator view of cognition. According to this view knowledge is receptive, as if the world causes in us a representation, or model, of reality. But the assumption that the cognitive enterprise is completed once we have a theory, or model, of our study object, is deeply mistaken. Cognition is fundamentally active. It is a feature of our organic interactions with and in our environment. Knowing, experimenting and acting go hand in hand, in iterative cycles of problem solving.
- Doing and undergoing. Every action is a reaction, a doing as well as an undergoing, a link in an endless process of intertwining interactions.
- Inner and outer. Our boundary as organism is fundamentally porous. We cannot survive without metabolism. Since human beings are essentially social and linguistic organisms, there is much more than merely physical stuff that goes in and comes out. The inner and the outer form an intimate, multi-layered, meaningful continuum, which will be obvious for any studying brain seriously studying brains.

Let us elaborate on two features of pragmatism to give some indication of how neuropragmatism might be a promising approach of neurometaphysics: (1) the organic way in which humans interact with their environment and (2) their activity as continuously learning in collaboration.

### 4.1. Organic interaction

Most basically for Dewey is the recognition that we are organisms (Dewey, 1916, 1922, 1929, 1938). This is also crucial for the second and third generation of neuropragmatists identified by Solymosi (2011), Dennett (1987), Clark (1997), Flanagan (1998), Thompson (2007), and Noë (2009). We are continuous with nature, with everything that lives, and so are our intelligence, our minds, our values, arts, laws, religion, science, and every other imaginable cultural achievement. We interact with our environment: continuously and organically, individually and collectively, emotionally and rationally, volitionally and cognitively. Science is one of the ways in which we interact, as natural organisms, with our environment. It may be our most sophisticated way, but we should not be fooled into believing that its ultimate product will be a true and objective theory of what there really is. Such a conception of science would merely reveal that we are the victims of our own gullibility to be taken by the aforementioned misleading dichotomies. Pragmatism precisely warns against this by emphasizing that science is an activity, a practice. Science is one way of solving problems, experimentally, constructing and positing, as Cassirer argued, out of what we measure the reality of what is measured (Cassirer, 1910, 1944). But measuring and positing what is measured, is by far not the only and most of the time not even the best way of interacting with our environment (Biesta, 2010).

From the pragmatist point of view it is actually rather misguided to think of science as a self-standing mode of interaction with our environment. Science should better be conceived of as one dimension of a multidimensional engagement with our environment, such as in the proposal of Neurotech^EU^ in which empirical and clinical neuroscience constitute just one of eight dimensions (NeurotechEU, 2023). Neuroscience, as part of neurotechnology, may nevertheless be particularly interesting for human self-understanding. After all, neuroscience seeks to investigate the dynamics of the nervous system, the inner structure that most clearly, and most sophisticatedly, manifests our capacity to adapt ourselves to our environment, in ways that are both assimilating and accommodating, ways that are intrinsically social, temporal and intelligent. We anticipate and try out, we plan, design and experiment, we build and re-build our environment, as well as ourselves – and so does our brain, mirroring on the inside what we as an organism do on the outside.

Grasping the fundamentally relational nature of our existence as socially intelligent organisms attuned to the affordances of both nature and culture, outer and inner, is the task of neurometaphysics. And the promise of neuropragmatism consists in the appreciation of this task as the task of grasping what is happening, both as doing and as undergoing, when studying brains study brains. The emphasis on the act of studying suits Dewey’s understanding of the fundamental importance of education for the human mode of existence, a mode characterized by lifelong learning.

### 4.2. Continuous learning

Living organisms are capable of self-organisation (Maturana and Varela, 1980; Varela, 1997). This is as true of amoebae, as it is true of trees, reptiles, mammals, and, thus, men. Self-organisation requires a permanent, flexible interaction with one’s environment driven by a concern for the survival of one’s integrity. We should be careful not to interpret this characterization too anthromorphically, but the purposefulness of *autopoietic* organisms seems rather fundamental. Living organisms are active, concerned, and dynamic, taking care of their existence in and over time (Di Paolo, 2005). The neuroplasticity of our brain is obviously key to this human mode of self-organisation, and activity, concern and dynamics are clearly well-known features of the brain’s plasticity.

From a pragmatist point of view the neuroplasticity of the brain is best understood as a form of continuous learning, of adapting to an everchanging environment, developing habits, reviewing and changing existing habits, and reinforcing confirmed habits. In these learning processes the inner and the outer are continuously two sides of one and the same coin. Our cognitive systems are dynamically adaptive to the interactions between organism and environment, developing improved habitual efficiency, such that, in Dewey’s words, “a living organism and its life processes involve a world or nature temporally and spatially ‘external’ to itself but ‘internal’ to its functions” (Dewey, 1925, p. 278). Notably, Dewey’s account here aligns with the recent neuroscientific (re)turns to predictive processing/coding and active inference (e.g., Friston et al., 2017), or neurological function as implied by the Free Energy Principle framework (Friston, 2010), even though these *still* maintain a Cartesian inner-outer distinction through positing internal representations.

Importantly, we are not alone. We are a social species. Survival would be completely impossible for us if not for the care and devotion of others who raise us, who educate us, who welcome us in their world and familiarize us with their ways of adapting to their environment (Sterelny, 2003). This social interdependency is crucially characteristic of human existence and it adds new dimensions to the inner and the outer, to the myriad ways in which our existence is literally *ours* (Tuomela, 2003). Our boundaries are fluid and porous. Our thoughts are ours in a significant way, not merely mine or yours, but *ours*. Our thoughts are informed by our language, articulated with the help of words that are no-one’s words in particular but ours in a, for Dewey, deeply democratic way. Conceptual frameworks are social achievements and so are our minds, as Dewey argued following his colleague and friend Mead (1934).

The social dimension of our existence naturally points out the importance of education as a fundamental mode of co-existence, given that we are, by nature, continuously learning – from our own endeavours, from our environment, from one another. Dewey’s influence in the philosophy and practice of education has been dominant, ever since his first publications (Siegel et al., 2018). There is a risk, too, in this, as there is a certain tendency to associate education with children and with the first phase of human life, which might incline us to misunderstand Dewey’s emphasis on continuous learning as key to organic life. The word ‘study’, therefore, might help to prevent this misunderstanding. Study is not merely what students do, as if in an attempt to complete their learning trajectory. Study is what researchers do, and what we do, all of us, common people, when we are challenged by changes in our environment, new happenings we did not anticipate. Study is what we do when we try to understand how to successfully adapt to our environment, when we try to solve our problems, when we experiment, develop and execute plans and assess steps undertaken. Study is our human mode of being. And in the slightly mistaken *pars pro toto* language of Neurotech^EU^’s mission this means that we are most fundamentally studying brains studying brains.

## 5. Conclusion

Neurometaphysics is an appropriate name for the attempt to study the nature and structure of our nervous system as most basically a relational reality. Our brains are not things, not substances. They are continuous dynamic processes of enabling the successful adaptive interaction between ourselves as intelligent organisms and our natural and social environment as an *Umwelt*, a field of supportive affordances.

We have argued that the legacy of an endemic Cartesianism threatens progress in neurometaphysics and by extension in neuroscience more broadly for – at least – two reasons. Neuroscientists might falsely assume that their subject matter consists of brains that can in principle be studied in isolation. And they might falsely assume that our agency as intelligent human beings is primarily a matter of deliberation, of making decisions, consciously and rationally or impulsively and emotionally. These assumptions might delude neuroscientists to overlook the importance of habits for understanding the work brains do in the world, as well as to underestimate this world’s role in acquiring and sustaining these habits.

We have sketched the promise of neuropragmatism, of John Dewey’s significance for the neurometaphysical enterprise. We have identified a range of dichotomies neuroscience should clearly steer away from and we have highlighted two features of pragmatism that Neurotech^EU^ might embrace to make sense of the larger picture in which neurotechnology has a part to play for humankind’s attempt to self-organize the continuous flourishing of human life. Our life is, firstly, a matter of socially intelligent organic interaction. And it requires, secondly, continuous learning, the never-ending capacity to adapt ourselves to an ever-changing environment.

We have neglected further issues, the most important one of which imposes itself in this last paragraph. Contemporary humankind lives in the Anthropocene (Crutzen, 2006). We have adapted our natural environment to our modern, capitalist ambition of never-ending economic growth. We seem to have forgotten that adaptation is a reciprocal relationship, embracing both assimilation and accommodation. It is time for us to accommodate, to take care of our environment before it is too late, before, that is, our environment will prove itself incapable of providing further support for our destructive exploitation. If we want neurotechnology to be a success – i.e. a success as a human endeavour – we shall have to prove our plasticity and our capacity for continuous learning, by finding the appropriate means to care for our environment and to solve the gigantic climate challenges: fossil energy, metal mining, bio industry, and soil exhaustion, and the related social challenges of democracy, poverty, and inequality. For the brain is indeed the most miraculous and wonderful product of natural evolution. But if it fails to solve these climate challenges, it will prove itself, unfortunately, not to be “marvellously effective in surviving in complex, partially unknown environments” (NeurotechEU, 2023) after all.

## Author contributions

FO contributed in thinking, developing, and writing. JB wrote the first draft of the article. All authors contributed to the article and approved the submitted version.

## Conflict of interest

The authors declare that the research was conducted in the absence of any commercial or financial relationships that could be construed as a potential conflict of interest.

## Publisher’s note

All claims expressed in this article are solely those of the authors and do not necessarily represent those of their affiliated organizations, or those of the publisher, the editors and the reviewers. Any product that may be evaluated in this article, or claim that may be made by its manufacturer, is not guaranteed or endorsed by the publisher.

## Footnotes

^1^https://theneurotech.eu/mission-vision/
