# Corrigendum: Studying Brains. What could neurometaphysics be to NeurotechEU?

**DOI:** 10.3389/fnins.2023.1245835

**Published:** 2023-07-18

**Authors:** Jan Bransen, Freek Oude Maatman

**Affiliations:** ^1^Philosophy Programme, Behavioural Science Institute, Faculty of Social Sciences, Radboud University, Nijmegen, Netherlands; ^2^Radboud Teaching and Learning Centre, Radboud University, Nijmegen, Netherlands; ^3^Department of Philosophy, Groningen University, Groningen, Netherlands

**Keywords:** Cartesianism, Dewey, continuous learning, metaphysics, neurometaphysics, neuropragmatism, relational ontology

In the published article, there was an error in the article title. Instead of “Studying brains what could neurometaphysics be to NeurotechEU?” it should be “Studying Brains. What could neurometaphysics be to NeurotechEU?”

The authors apologize for this error and state that this does not change the scientific conclusions of the article in any way. The original article has been updated.

